# crossNN is an explainable framework for cross-platform DNA methylation-based classification of tumors

**DOI:** 10.1038/s43018-025-00976-5

**Published:** 2025-06-06

**Authors:** Dongsheng Yuan, Robin Jugas, Petra Pokorna, Jaroslav Sterba, Ondrej Slaby, Simone Schmid, Christin Siewert, Brendan Osberg, David Capper, Skarphedinn Halldorsson, Einar O. Vik-Mo, Pia S. Zeiner, Katharina J. Weber, Patrick N. Harter, Christian Thomas, Anne Albers, Markus Rechsteiner, Regina Reimann, Anton Appelt, Ulrich Schüller, Nabil Jabareen, Sebastian Mackowiak, Naveed Ishaque, Roland Eils, Sören Lukassen, Philipp Euskirchen

**Affiliations:** 1 Department of Experimental Neurology, Charité – Universitätsmedizin Berlin, corporate member of Freie Universität Berlin and Humboldt-Universität zu Berlin, Berlin, Germany; 2 Center of Digital Health, Berlin Institute of Health at Charité – Universitätsmedizin Berlin, Berlin, Germany; 3 Department of Biology, Faculty of Medicine and Central European Institute of Technology, Masaryk University, Brno, Czech Republic; 4 Department of Pediatric Oncology, University Hospital Brno, Faculty of Medicine, Masaryk University, Brno, Czech Republic; 5 Department of Neuropathology, Charité – Universitätsmedizin Berlin, corporate member of Freie Universität Berlin and Humboldt-Universität zu Berlin, Berlin, Germany; 6 German Cancer Consortium (DKTK), Partner Site Berlin, German Cancer Research Center (DKFZ), Heidelberg, Germany; 7 Vilhelm Magnus Laboratory, Institute for Surgical Research, Department of Neurosurgery, Oslo University Hospital, Oslo, Norway; 8 Institute for Clinical Medicine, Faculty of Medicine, University of Oslo, Oslo, Norway; 9 Dr. Senckenberg Institute of Neurooncology, Goethe University Frankfurt, University Hospital, Frankfurt, Germany; 10 Department of Neurology, Goethe University Frankfurt, University Hospital, Frankfurt, Germany; 11 Frankfurt Cancer Institute (FCI), Goethe University Frankfurt, Frankfurt, Germany; 12 Neurological Institute (Edinger Institute), Goethe University Frankfurt, University Hospital, Frankfurt, Germany; 13 German Cancer Consortium (DKTK), Partner Site Frankfurt, German Cancer Research Center (DKFZ), Heidelberg, Germany; 14 University Cancer Center (UCT) Frankfurt, Goethe University Frankfurt, University Hospital, Frankfurt, Germany; 15 Department of Neuropathology, LMU München, Munich, Germany; 16 Institute of Neuropathology, University of Münster, Münster, Germany; 17 Department of Pathology and Molecular Pathology, University Hospital and University of Zurich, Zurich, Switzerland; 18 Institute of Neuropathology, University Hospital and University of Zurich, Zurich, Switzerland; 19 Department of Pediatric Hematology and Oncology, University Medical Center Hamburg-Eppendorf, Hamburg, Germany; 20 Research Institute Children’s Cancer Center Hamburg, Hamburg, Germany; 21 Institute of Neuropathology, University Medical Center Hamburg-Eppendorf, Hamburg, Germany; 22 Department of Neurology, Charité – Universitätsmedizin Berlin, corporate member of Freie Universität Berlin and Humboldt-Universität zu Berlin, Berlin, Germany

**Keywords:** Cancer, Machine learning, CNS cancer, Cancer of unknown primary, DNA methylation

## Abstract

DNA methylation-based classification of (brain) tumors has emerged as a powerful and indispensable diagnostic technique. Initial implementations used methylation microarrays for data generation, while most current classifiers rely on a fixed methylation feature space. This makes them incompatible with other platforms, especially different flavors of DNA sequencing. Here, we describe crossNN, a neural network-based machine learning framework that can accurately classify tumors using sparse methylomes obtained on different platforms and with different epigenome coverage and sequencing depth. It outperforms other deep and conventional machine learning models regarding accuracy and computational requirements while still being explainable. We use crossNN to train a pan-cancer classifier that can discriminate more than 170 tumor types across all organ sites. Validation in more than 5,000 tumors profiled on different platforms, including nanopore and targeted bisulfite sequencing, demonstrates its robustness and scalability with 99.1% and 97.8% precision for the brain tumor and pan-cancer models, respectively.

## Abstract (web summary)

Yuan et al. present crossNN, an explainable machine learning tool to classify tumors based on DNA methylation data from several platforms with different epigenome coverage and sequencing depth, benchmark against other models and validate externally.

## Main

DNA methylation has an important role in the regulation of gene expression and cell type differentiation^1,2^. Patterns of 5-methylcytosine (5mC) define physiological cell states, but have also been linked to many human diseases, including cancer^3–5^. In medicine, epigenome-wide patterns of 5mC can be exploited for disease classification^6^. In particular, DNA methylation-based classification of tumors has emerged as a powerful conceptual and diagnostic tool both for establishing a clinical diagnosis and for investigating the molecular taxonomy of cancer^7–9^. Indeed, the classification of central nervous system (CNS) tumors has been embraced by the World Health Organization (WHO)^10^ with profound impact on routine diagnostic workup^4,5^. Moreover, integrated histo-molecular classification of brain tumors has been shown to refine histological diagnosis, with reclassification in about 12% of cases^8^. Most implementations of diagnostic assays rely on the generation of methylation profiles using hybridization microarray and supervised classification against a well-annotated reference dataset^11,12^, which has become a widely accepted diagnostic approach in adult and pediatric neuro-oncology^8,13–15^.

However, several methods for probing the 5mC methylome have been developed and benchmarked, each providing information on DNA methylation in different target regions and at different levels of resolution^16^. For example, whole-genome bisulfite sequencing (WGBS) has long been seen as a gold standard in providing the most comprehensive DNA methylation map at single-base resolution^17^. However, WGBS is expensive and demands substantial quantities of input DNA. Moreover, sequenced reads often lack useful methylation information^18^. Targeted methylation sequencing (targeted methyl-seq) using restriction enzymes or, more recently, hybridization capture for enrichment has gained widespread popularity for cost-efficient targeted capture^19,20^. Microarray-based technologies, such as Infinium HumanMethylation450 (Infinium 450K) and Infinium HumanMethylation850 (MethylationEPIC, EPIC) have also been widely used to survey specific genomic loci across the genome using bespoke probes^21^. More recently, third-generation sequencing techniques have allowed base modifications from natural DNA to be inferred. We and others demonstrated the suitability and robustness of low-coverage whole-genome nanopore sequencing in clinical application for accurate, rapid and cost-efficient DNA methylation-based classification of brain tumors^22,23^. However, the commonly aimed for ultralow sequencing depth and coverage leads to mostly binary methylation information (instead of beta values) of a random subset of the approximate 30 million CpG sites in the genome^23^.

All these methods deliver highly concordant results; however, different genomic coverage and depth have so far required different assay-specific approaches to classification^24^. Several machine learning algorithms have been used for this task, but are mainly restricted to single-platform data or fixed-feature spaces, for example, the most commonly used random forest (RF) model for use with microarray data^8^. Previously, we proposed ad-hoc RFs that can bridge the gap between low-coverage nanopore sequencing data and microarray reference data at the expense of training a new ad-hoc model for each unknown sample; however, this is time-consuming, computationally expensive and introduces non-comparability between these patient-specific models^23^. Recently, a neural network (NN)-based model was proposed, which uses sparse data to predict brain tumor classes^25^. A precise model that can predict brain tumor classes across platforms is still urgently needed.

In this study, we propose crossNN, a unified NN-based framework trained on fixed reference data that handles variable and sparse feature datasets for prediction. The model enables instantaneous predictions from methylation profiles generated by multiple platforms, including WGBS, targeted methyl-seq, low-coverage nanopore whole-genome sequencing (WGS) and several microarray platforms (Illumina 450K, EPIC, EPICv2). At the same time, the lightweight scalable architecture allows for rapid retraining and cross-validation (CV) for the rapidly emerging landscape of cancer reference atlases.

## Results

### Model development and workflow

**Fig. 1:**
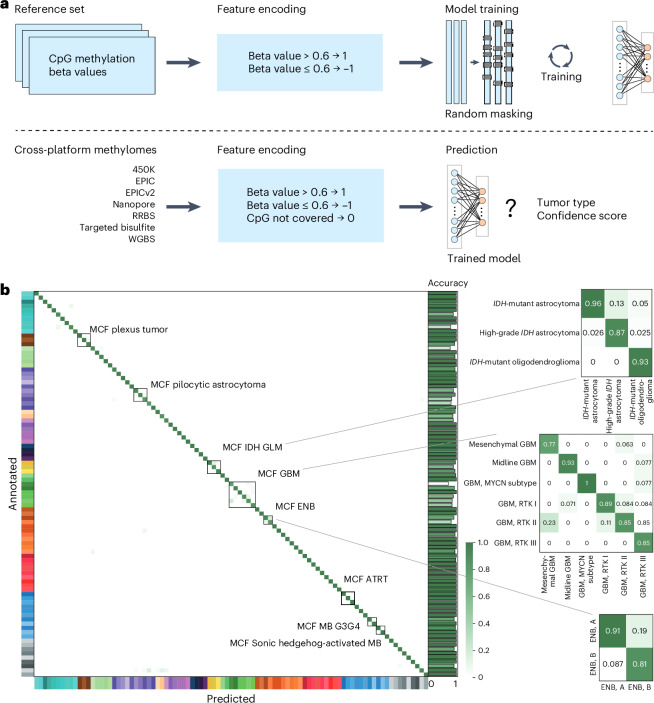
crossNN model architecture, training and CV. **a**, Overview of the model architecture. **b**, Heatmap of confusion matrix in fivefold CV. ATRT, atypical teratoid/rhabdoid tumor; ENB, esthesioneuroblastoma; MB, medulloblastoma; MB G3G4, MB group 3 and group 4; RRBS, reduced representation bisulfite sequencing; RTK, receptor tyrosine kinase (I, II and III). Source data

The crossNN model architecture (Fig. 1a) relies on a perceptron, implemented as a single-layer NN using PyTorch (see ‘Model training’ in Methods). The network architecture consists of an input layer and an output layer with the two layers being fully connected without bias; this means that the model will capture the linear relationship between the input CpG sites and methylation classes (MCs). For training, we used the Heidelberg brain tumor classifier v11b4 reference dataset, consisting of the methylation profiles of 2,801 samples from 82 tumor types and subtypes (MCs) and nine non-tumor control classes, generated using Illumina 450K microarrays^8^. The feature space of the training dataset is fixed, given the array probe set, and mainly covers CpG sites in CpG islands and promoter regions.

During preprocessing and for cross-platform normalization, CpG sites in the training dataset were binarized using an empirically determined beta value threshold of 0.6 (ref. ^23^). Thereafter, uninformative probes were removed (see 'Feature selection' in Methods), resulting in a total of 366,263 binary features.

To enable tumor classification using different platforms for methylome profiling with varying or sparse epigenome coverage, the model was trained with randomly and repeatedly masked input data. Masked CpG sites during training were encoded as zero, unmethylated sites as −1 and methylated probes as 1. The model was then trained using the randomly resampled and (−1,1)-encoded binary training dataset. For prediction from methylation profiles from different platforms, methylated allele frequencies at CpG sites were equally binarized and missing features encoded as zero.

**Extended Data Fig. 1:**
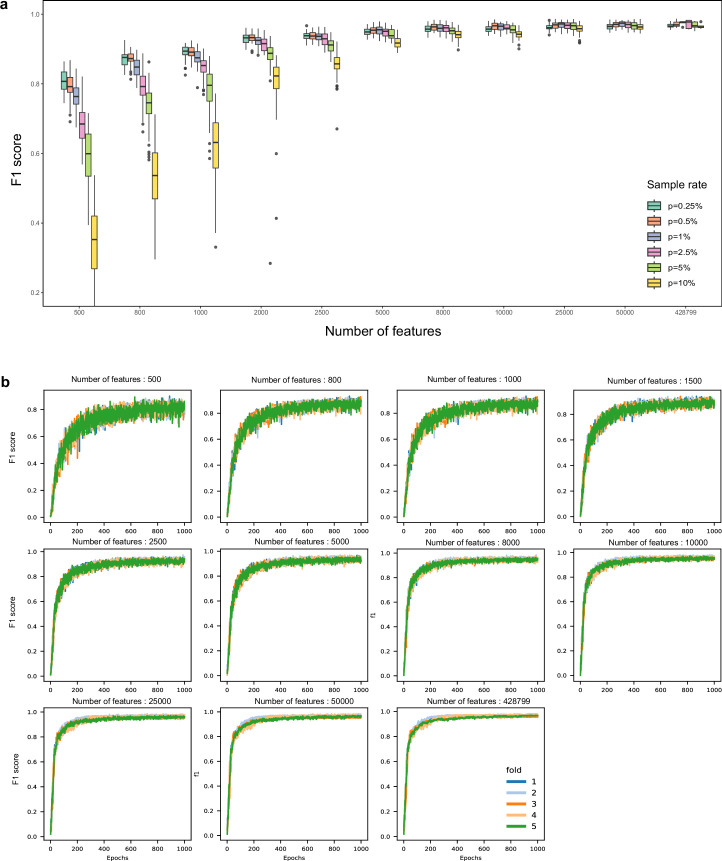
Identification of optimal sampling rate and number of epochs for training crossNN. (**a**) Comparison of F1 score for various sampling rates via 5-fold cross validation (5xCV) with different numbers of features. Each box plot indicates median F1 score (center line), inter-quartile range (box) and 1.5fold interquartile range (whiskers). Outliers are indicated by dots. Downsampling and 5xCV were performed 10 times for the given number of features. (**b**) F1 score vs. number of epochs in 5xCV for a given number of features that the training set has been downsampled to. Source data

Critical hyperparameters that were optimized included the masking rate p and the number of epochs e, which is proportional to how many times each sample is resampled. Using a grid search approach, a masking rate of 99.75% (Extended Data Fig. 1a) and 1,000 epochs (Extended Data Fig. 1b) were selected to train the final model.

### Evaluation of model performance

**Extended Data Fig. 2:**
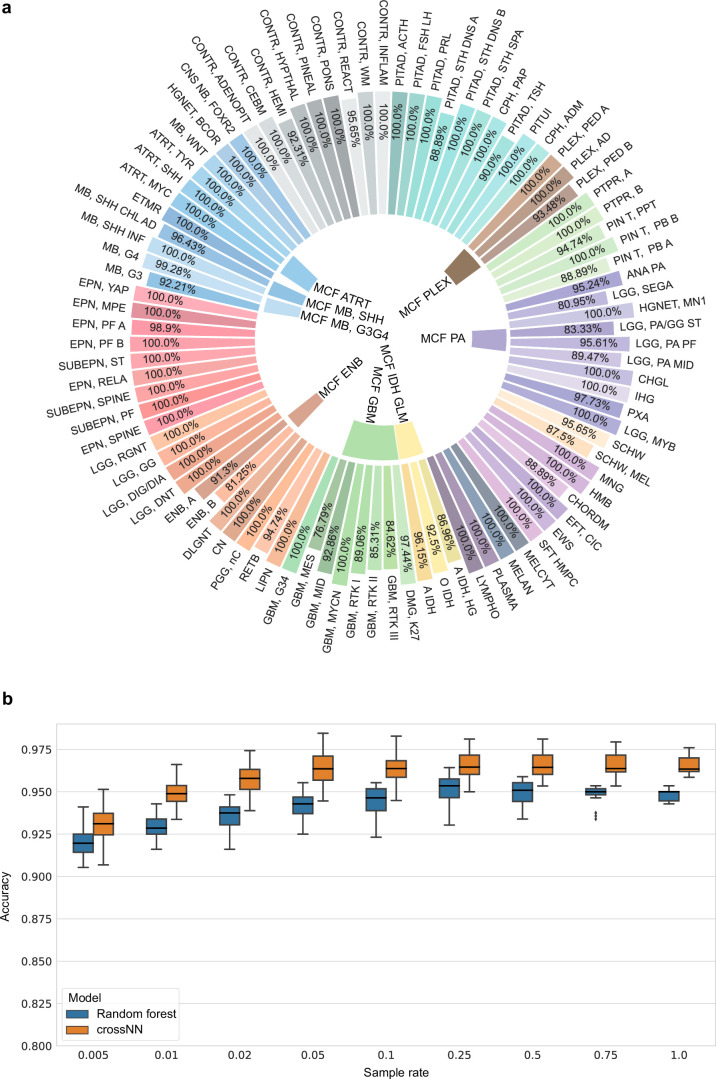
Model performance in 5-fold cross validation (CV) of the 450 K training set. Model performance in 5-fold cross validation (5xCV) of the 450 K training set. (**a**) Accuracy for each individual methylation class and methylation class family (MCF) during 5-fold CV. (**b**) Overall accuracy of the crossNN model in 5xCV of the training set. Validation folds were subsampled at the indicated rate to simulate sparse methylomes. Random sampling and 5xCV were repeated ten times at each sample rate. Box plots indicate median accuracy (center line), inter-quartile range (box) and 1.5fold interquartile range (whiskers). Outliers are indicated by dots. Source data

First, model performance was validated using fivefold CV in the training dataset. Overall accuracy was 96.11 ± 0.86% across all folds at the MC level (Fig. 1b and Extended Data Fig. 2a). Tumor classes in the same MC family (MCF) are closely related and misclassifications inside an MCF will usually not have clinical impact. Indeed, most misclassifications were observed in the MCF (Fig. 1b). Therefore, at the MCF level, prediction accuracy reached 99.07 ± 0.21%. In comparison, ad-hoc RF models for each subsampled feature dataset reached lower accuracy both at the MC and MCF levels (94.93 ± 0.88% and 97.89 ± 0.60%, respectively).

To further test our model’s performance using samples with different coverages of the CpG sites, the microarray samples on the test folds were subsampled with different sampling rates from 0.5% to 100%; for each sampling rate, we repeated this process randomly ten times. Our model showed robust performance, with high average accuracy in fivefold CV with different sampling rates from 0.5% to 75%, outperforming ad-hoc RFs (Extended Data Fig. 2b).

### Independent CV in different platforms

Next, we validated the final model in independent cohorts generated on different microarray and sequencing platforms. We assembled a validation cohort of 2,090 patient samples generated on Illumina 450K (*n* = 610), EPIC (*n* = 554) and EPICv2 (*n* = 133) microarrays, as well as nanopore low-pass WGS (*n* = 415 with R9 chemistry; *n* = 129 with R10 chemistry), Illumina targeted methyl-seq (*n* = 124) and Illumina WGBS (*n* = 125) (Supplementary Table 1). The validation dataset covered 62 different brain tumor types (WHO integrated diagnosis^10^), reflecting 67 of the 82 MCs in the training dataset.

**Fig. 2:**
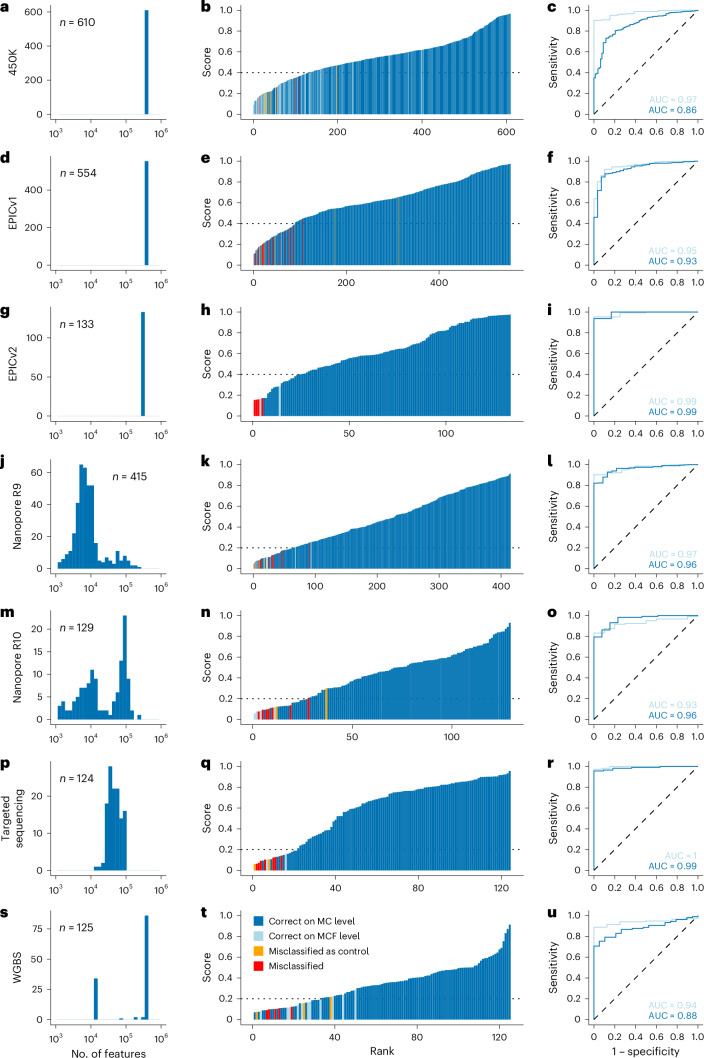
Classification results in the 450K, EPIC/EPICv2, nanopore, targeted methyl-seq and WGBS validation cohorts. **a**,**d**,**g**,**j**,**m**,**p**,**s**, Predictions for 2,090 samples are shown (450K *n* = 610 (**a**), EPICv1 *n* = 554 (**d**), EPICv2 *n* = 133 (**g**), nanopore R9 *n* = 415 (**j**), nanopore R10 *n* = 129 (**m**), targeted sequencing *n* = 124 (**p**), WGBS *n* = 125 (**s**)). The distribution of the number of CpG features used as input to the crossNN model is shown. **b**,**e**,**h**,**k**,**n**,**q**,**t**, Waterfall plots of cohorts with samples ranked according to the confidence score. The dashed lines indicate platform-specific cutoff values chosen based on fivefold CV. **c**,**f**,**i**,**l**,**o**,**r**,**u**, Receiver operator characteristics of confidence scores regarding the correct classification on MC versus MCF level.

Depending on the assay, the distribution of the number of CpG features used for prediction varied by two orders of magnitude (Fig. 2a–s). Nevertheless, we achieved a high overall accuracy of 0.91 and an area under the curve (AUC) of 0.95 (ranging from 0.86 to 0.99 per platform for MC level classification; Fig. 2c–u). Again, most misclassifications occurred in the MCF (Fig. 2b–t). When aggregating scores to MCF level, overall accuracy was 0.96 and the mean AUC was 0.95 (ranging from 0.93 to 1 per platform; Fig. 2c–u).

**Extended Data Fig. 3:**
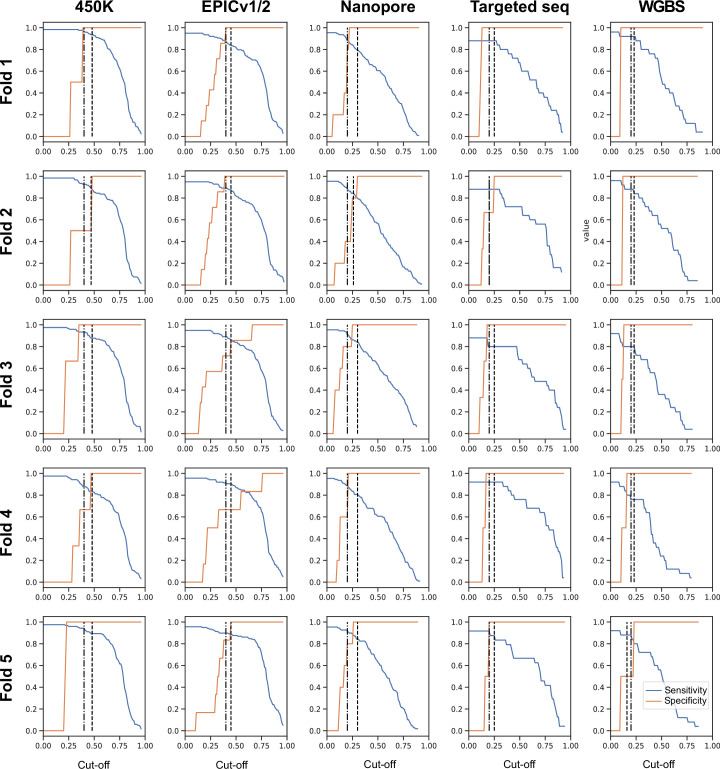
Identification of optimal platform-specific cut-off values for prediction scores of the brain tumor model. Plots show receiver operating characteristics (ROC) of MCF scores for individual folds in 5-fold cross-validation. Dashed vertical lines indicate Youden index, dashed-dotted lines indicate final chosen cut-off. MCF, methylation class family. Source data

However, essential to clinical application is the interpretation of classification results in the context of the confidence score. Therefore, we sought to establish cutoffs for diagnostic application. Because the distribution of prediction scores varied across platforms (Fig. 2b–t), we identified platform-specific diagnostic cutoffs for correct classification using per-platform fivefold CV. The optimal cutoff in each fold was determined by inspecting the Youden index of the receiver operating curve (ROC) (Extended Data Fig. 3). The range of optimal cutoffs was similar for the microarray and sequencing platforms. For simplicity, we selected a cutoff greater than 0.4 for all microarray platforms and greater than 0.2 for all sequencing platforms. This resulted in an overall precision of 0.98 on MC level and 0.99 on MCF level, respectively.

### Comparison to other algorithms

Next, we compared model and cutoff performance to our previously published ad-hoc RF approach^23^ and a recently published deep neural network (DNN), that is, Sturgeon DNN^25^. All approaches were developed to make predictions from sparse nanopore data, yet can be applied to any source of methylation data and use an identical training dataset.

Our shallow NN model was not inferior to ad-hoc RF and Sturgeon DNN regarding overall accuracy, and outperformed both approaches in terms of ROC characteristics of the prediction scores, especially precision (Table 1).

**Table 1:** Comparison of the crossNN model to ad-hoc RFs^23^ and the Sturgeon DNN approach^25^

Cohort	Number of cases	Metric	crossNN	Sturgeon 0.8	Sturgeon 0.95	Ad-hoc RF
450K	610	Accuracy	**0.979**	0.962	0.962	0.97
		Precision	**0.996**	0.973	0.988	0.972
		Sensitivity	0.93	0.861	0.792	**0.966**
		AUC	**0.973**	0.892	0.892	0.921
EPICv1	554	Accuracy	0.948	0.955	0.955	**0.966**
		Precision	**0.99**	0.963	0.967	0.971
		Sensitivity	0.894	0.944	0.908	**0.96**
		AUC	**0.953**	0.773	0.773	0.884
EPICv2	133	Accuracy	0.97	**1**	**1**	0.985
		Precision	**1**	**1**	**1**	0.992
		Sensitivity	0.895	0.977	0.94	**0.985**
		AUC	0.986	NaN	NaN	0.992
Nanopore R9	415	Accuracy	**0.964**	0.925	0.925	0.937
		Precision	**0.99**	0.964	0.973	**0.99**
		Sensitivity	**0.908**	0.824	0.61	0.718
		AUC	**0.967**	0.843	0.843	0.917
Nanopore R10	129	Accuracy	**0.922**	0.884	0.884	0.899
		precision	0.965	0.954	0.987	**1**
		sensitivity	**0.853**	0.791	0.581	0.674
		AUC	**0.931**	0.905	0.905	0.914
Targeted sequencing	124	Accuracy	**0.895**	0.855	0.855	0.839
		Precision	0.991	0.994	**1**	0.99
		Sensitivity	**0.879**	0.806	0.726	0.766
		AUC	**0.997**	0.954	0.954	0.958
WGBS	125	Accuracy	**0.936**	0.808	0.808	0.88
		Precision	**0.991**	0.892	0.922	0.979
		Sensitivity	**0.848**	0.616	0.432	0.736
		AUC	**0.94**	0.79	0.79	0.918
Overall	2,090	Accuracy	**0.956**	0.935	0.935	0.946
		Precision	**0.991**	0.963	0.978	0.947
		Sensitivity	**0.901**	0.861	0.757	0.873
		AUC	**0.953**	0.865	0.865	0.9

For each model, MCF-level raw accuracy before the application of cutoffs, precision with platform-specific cutoffs and AUC of the ROC for the (calibrated) score to predict the correct classification are given. For crossNN, the following cutoffs, as derived above, were used: microarray > 0.4; crossNN nanopore/targeted methyl-seq/WGBS > 0.2. Published validated cutoffs were used for ad-hoc RF and the Sturgeon DNN (ad-hoc RF > 0.15; Sturgeon DNN > 0.8 or > 0.95, respectively). NaN, not a number. Bold indicates row-wise maximum values.

### Interpretability of the model

Our model’s architecture facilitates interpretability by capturing the linear relationships between CpG probes and tumor classes or subclasses. Thus, the weights of the edges connecting the input CpG features and the output layer can be interpreted as indicators of feature importance, offering insights into the relevance of individual CpG probes in the classification of specific tumor types: each CpG feature is assigned a positive or negative weight for each tumor type. Positive weights indicate that if a given CpG site is methylated, the sample is more likely to match the corresponding tumor type, and vice versa.

**Fig. 3:**
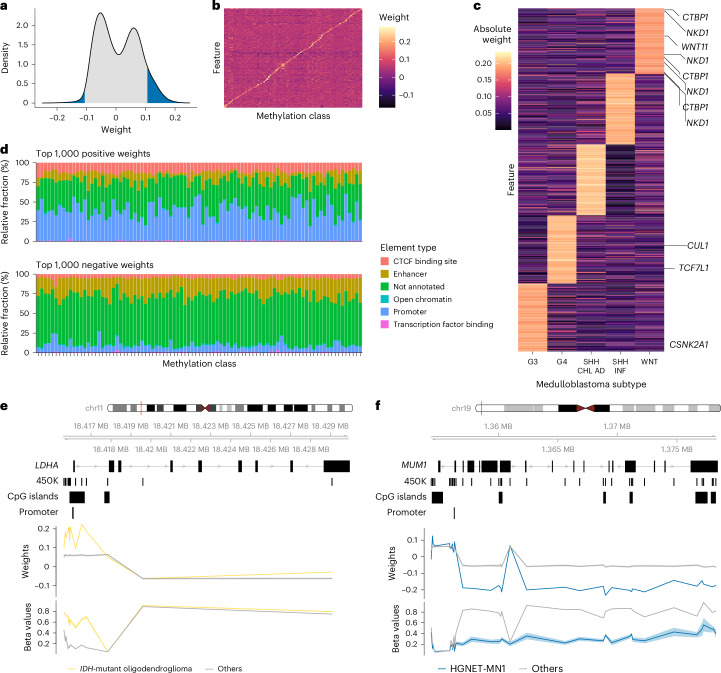
Interpretability of the model. **a**, Typical bimodal distribution of feature weights. As an example, the distribution of feature weight values (*n* = 366,263 features) for the MC oligodendroglioma, *IDH*-mutant and 1p/19 code-deleted (*IDH*-mutant oligodendroglioma) are shown. The blue shading of the AUC indicates the top 5% of features ranked according to absolute weight. **b**, Heatmap illustrating the methylation levels (beta value) of the top ten CpG sites per MC (*n* = 91 classes), ranked according to feature weight in the final prediction model. For illustration, only features with a positive weight were considered during ranking. **c**, Clustered heatmap of the top 200 features ranked according to the absolute weight for each of the MB subtypes. Genes associated with Wnt signaling according to Gene Ontology terms are annotated. **d**, Annotation and summary of regulatory elements overlapping the top 1,000 positively and negatively weighted features per MC (*n* = 91 classes). **e**,**f**, Importance of class-specific features with respect to genomic context. **e**, The differential promoter methylation of *LDHA* was identified using feature ranking as a distinct feature of oligodendroglioma. The average beta values from oligodendrogliomas (*n* = 80 cases) versus all other reference samples (*n* = 2,721 cases) are shown. **f**, Conversely, the *MUM1*/*PWWP3A* gene was identified as a marker gene for the MC ‘high grade neuroepithelial tumors with MN1 alterations’ *(HGNET*-*MN1)* using the ranking of feature weights aggregated at the gene level. Differential hypomethylation was observed in the gene body, but not in a proximal CpG island (lower track). The average beta values from *HGNET*-*MN1* (*n* = 21 cases) versus all other reference samples (*n* = 2,780 cases) are shown. AD, adolescent; CHL, child; INF, infantile; SHH, Sonic hedgehog. Source data

The absolute value of the weight reflects the importance of a given CpG site in predicting the associated tumor type. For each tumor type, CpG sites with top positive and negative weights (Fig. 3a,b) are differentially methylated between tumor (sub)types, which can be helpful to reveal the biological mechanisms underlying tumor type identity, such as cell of origin, and discover potential biomarkers. We first investigated whether the model could identify known associations with signaling pathways. Indeed, important CpG sites corresponding to genes involved in Wnt signaling were enriched in the Wnt-activated subtype of medulloblastoma (Fig. 3c), linking classification features to pathogenetically relevant biological processes.

Next, we investigated the association of feature importance with regulatory elements. We separately annotated the top 1,000 most important negative and positive feature weights for each tumor type in the crossNN model. Across MCs, gene promoters were enriched among positive weights while enhancers were enriched among negatively correlated features (Fig. 3d).

We then studied the differential importance of CpG sites in a given gene locus. For example, CpG sites in the lactate dehydrogenase A (*LDHA*) promoter were identified among the top features relevant for oligodendroglioma, as indicated by positive weights and hypermethylation of the nearby CpG island, while CpG sites in the gene body were not differentially methylated (Fig. 3e). In contrast, important CpG sites in the PWWP domain containing 3A, DNA repair factor (*PWWP3A*, commonly known as *MUM1)* gene locus for the prediction of MC high-grade neuroepithelial tumor with MN1 alteration (HGNET-MN1) were located in the gene body. In accordance with the negative weight of most features, the *MUM1* gene body was hypomethylated while promoter methylation was not informative (Fig. 3f). The HGNET-MN1 class corresponds to a new tumor type recently endorsed by the 2021 WHO classification^10^ as astroblastoma, MN1-altered^10^ and mRNA expression of *MUM1* has previously been identified as marker gene for HGNET-MN1^26^.

In summary, the feature importance revealed by the model sheds light on the functional importance of individual (marker) genes and can quantify the positional importance of epigenetic modifications in a gene’s structure. As such, the crossNN model architecture uses epigenomic features for classification that can be individually linked to transcriptional regulation and cellular signaling, making the models fully explainable.

### Pan-cancer classification

**Fig. 4:**
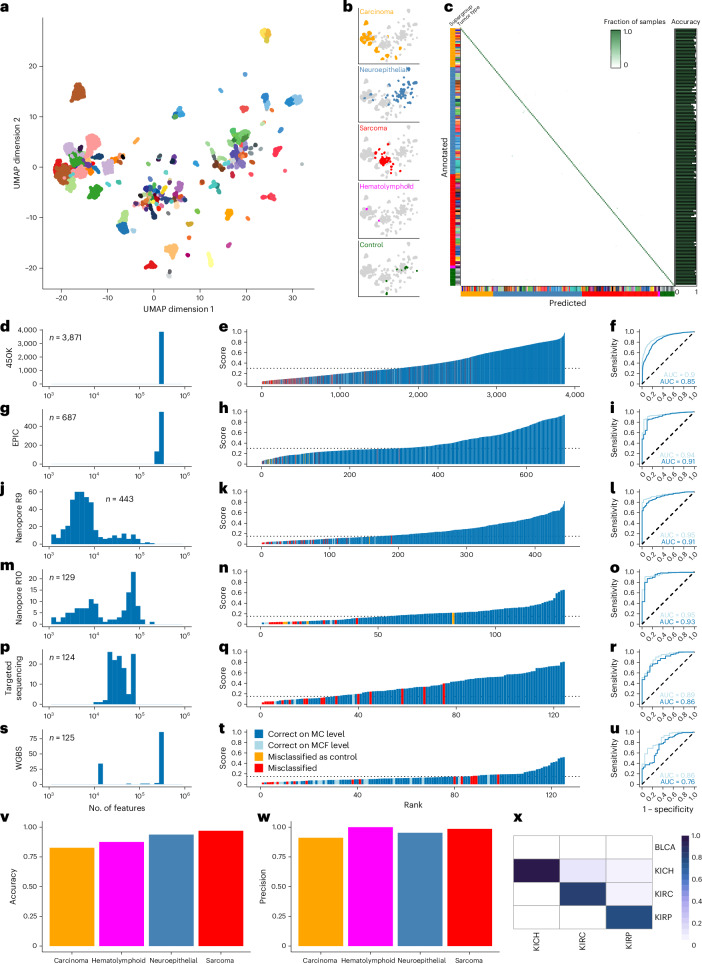
Validation of a crossNN pan-cancer classifier. **a**,**b**, Overview of the pan-cancer training dataset. Uniform manifold approximation and projection (UMAP) dimensionality reduction depicts the reference dataset of 8,382 reference tumors (**a**), including four major groups of tumors (**b**). **c**, Confusion matrix showing the internal validation of the crossNN pan-cancer model (*n* = 8,382 training samples). **d**–**u**, Independent validation of the model across different platforms. **d**,**g**,**j**,**m**,**p**,**s**, Distribution of the number of CpG features used as input to the crossNN model: 450K (**d**), EPIC (**g**), nanopore R9 (**j**), nanopore R10 (**m**), targeted sequencing (**p**) and WGBS (**s**). **e**,**h**,**k**,**n**,**q**,**t**, Waterfall plots of cohorts with samples ranked according to confidence score. The dashed lines indicate platform-specific cutoff values chosen based on fivefold CV. **f**,**i**,**l**,**o**,**r**,**u**, Receiver operating characteristics of confidence scores regarding the correct classification on MC versus MCF level. **v**,**w**, Accuracy (**v**) and precision (**w**) in the validation cohort per major tumor group across all platforms (carcinoma *n* = 3,005, hematolymphoid *n* = 32, neuroepithelial *n* = 2,079, sarcoma *n* = 263 cases, respectively). **x**, Classification of renal cell carcinoma. The confusion matrix shows fractions relative to the total number of cases per subtype (kidney chromophobe renal cell carcinoma (KICH) *n* = 20, kidney renal clear cell carcinoma (KIRC) *n* = 107, kidney renal papillary carcinoma (KIRP) *n* = 86 cases, respectively). The columns indicate the ground truth, the rows indicate the crossNN predictions. BLCA, bladder urothelial carcinoma. Source data

To investigate the generalizability of the crossNN architecture, we next assembled a pan-cancer reference dataset to train a pan-cancer crossNN model. The training dataset consisted of 8,382 cases from 178 tumor types across most organ sites (Fig. 4a,b), assembled entirely from public data (Supplementary Table 2).

We used similar training parameters (masking rate 99.5%, 3,000 epochs) as for the brain tumor model. Internal validation (Fig. 4c) and fivefold internal CV showed an overall accuracy of 94.82 ± 0.06% on MC level and 97.61 ± 0.05% on MCF level, respectively.

**Extended Data Fig. 4:**
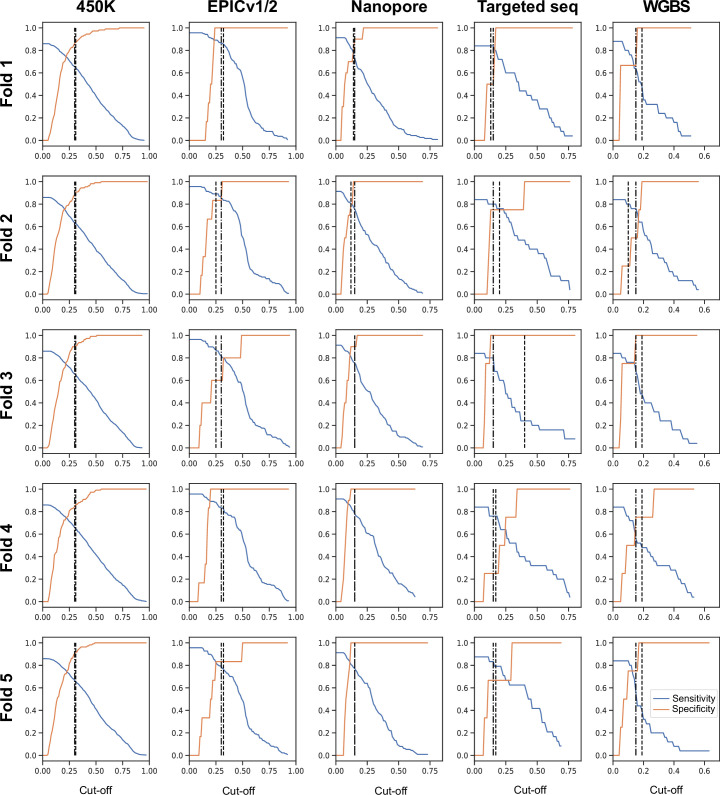
Identification of optimal platform-specific cut-off values for prediction scores of the pan-cancer model. Plots show receiver operating characteristics (ROC) characteristics of MCF scores for individual folds in 5-fold cross-validation. Dashed vertical lines indicate Youden index, dashed-dotted lines indicate final chosen cut-off. MCF, methylation class family. Source data

We next validated the model in 5,379 cases not seen before and generated on different microarray and sequencing platforms (Fig. 4d–u). Overall accuracy on MC and MCF level was 0.83 and 0.88, respectively. We again determined platform-specific cutoffs for the classification score using fivefold CV (Extended Data Fig. 4), with a classification score of more than 0.3 for the microarray and more than 0.15 for the sequencing platforms, respectively; precision was 0.98 (Table 2). Accuracy was lower in the subcohort of carcinomas compared to primary brain tumors, sarcomas and hematolymphoid malignancies (Fig. 4v); however, precision was high across all these major tumor types (Fig. 4w).

**Table 2:** Performance of the crossNN pan-cancer model in several cohorts

Cohort	Number of cases	Metrics	crossNN
450K	3,871	Accuracy	0.859
		Precision	0.975
		Sensitivity	0.654
		AUC	0.901
EPICv1	554	Accuracy	0.949
		Precision	0.962
		Sensitivity	0.926
		AUC	0.926
EPICv2	133	Accuracy	0.992
		Precision	0.992
		Sensitivity	0.977
		AUC	0.939
Nanopore R9	443	Accuracy	0.928
		Precision	0.997
		Sensitivity	0.772
		AUC	0.947
Nanopore R10	129	Accuracy	0.860
		Precision	0.989
		Sensitivity	0.713
		AUC	0.949
Targeted sequencing	124	Accuracy	0.847
		Precision	0.935
		Sensitivity	0.806
		AUC	0.895
WGBS	125	Accuracy	0.848
		Precision	0.941
		Sensitivity	0.640
		AUC	0.860
Overall	5,379	Accuracy	0.877
		Precision	0.978
		Sensitivity	0.691
		AUC	0.897

MCF-level raw accuracy before the application of cutoffs, precision with platform-specific cutoffs and AUC of the ROC curve for the classification score to predict the correct classification are given. For crossNN, the following cutoffs, as derived above, were used: microarray > 0.3; nanopore/targeted methyl-seq/WGBS > 0.15.

Misclassification was mainly observed among squamous cell carcinomas, which are known to share similar methylation (and gene expression) profiles across anatomic sites^9^. Therefore, we introduced a ‘squamous cell carcinoma superfamily’ MCF. Among high-confidence predictions, some recurrent misclassifications were observed. For example, papillary and clear cell renal carcinomas were frequently confused (Fig. 4x).

## Discussion

In this study, we present a simple and explainable machine learning framework that can accurately classify tumor entity using DNA methylation profiles obtained from different platforms and with different epigenome coverage and sequencing depth. It outperforms other deep and shallow machine learning models with respect to precision and simplicity, and computational requirements (for both training and prediction), while still being fully explainable. Validation in low-pass nanopore WGS, WGBS, targeted methyl-seq and microarray brain tumor cohorts demonstrates the robustness and scalability of the model. The architecture is highly scalable, as demonstrated by the training and validation of a pan-cancer classifier.

Mainly developed for sparse methylomes generated by ultralow-pass nanopore WGS, this pretrained model enables predictions in seconds, outperforming our previous ad-hoc RF implementation, which required time-consuming and computationally intense retraining for individual samples^23,27^. Immediate predictions greatly improve time-critical applications, such as intraoperative diagnostics. Compared to a recently published deep NN model^25^ trained on the same dataset, its performance is not inferior with respect to overall accuracy and is superior with respect to precision when applying diagnostic cutoffs on prediction scores, which is critical to ensure high specificity in clinical application. At the same time, the lightweight architecture allows rapid training on new reference datasets.

Despite using an NN architecture, the model maintains a simple linear structure, which limits overfitting and drastically increases the interpretability of the model. Feature importance guides biological and clinical interpretation of the model and facilitates marker gene detection in each tumor type. It also greatly facilitates the regulatory aspects of in vitro diagnostics supported by machine learning algorithms.

Importantly, the model is compatible with the EPICv2 microarray platform whose probe set is not downward-compatible and precludes the use of most versions of the original Heidelberg brain tumor classifier. We provide an intuitive web-based graphical user interface that allows users to upload methylation data and predict tumor entity instantaneously (https://crossnn.charite.de). Additionally, the model and source code are available for local deployment and integration with institutional workflows (https://gitlab.com/euskirchen-lab/crossnn).

Implementation of pan-cancer classifiers exemplifies the scalability of crossNN. It extends the scope application of DNA-methylation-based classification beyond brain tumors and will be particularly useful in the diagnostic workup of cancers of unknown primary.

Of note, we observed lower accuracy when validating our pan-cancer classifier in The Cancer Genome Atlas (TCGA) data compared to the meticulously curated validation cohorts available for brain tumors and sarcomas. Kidney tumor types were frequently confused, which might either indicate a weakness of the model or the shortcomings of current histological classification. In many challenging diagnostic scenarios, such as classification of primary brain tumors^8^, sarcomas^7^ and discrimination of lung versus head and neck squamous cell carcinomas^28^, such discrepancies between molecular and histological classification were largely resolved in favor of DNA-methylation-based classification.

Despite careful study design, the study has some limitations. First, we used binarization of methylated allele frequencies as a means for cross-platform normalization and feature encoding. However, using an empirically chosen global cutoff for binarization might be suboptimal for some MCs and might introduce bias. For tumor types with global hypomethylation or hypermethylation (such as pituitary tumors or isocitrate dehydrogenase (*IDH*)-mutant glioma (GLM), respectively) or low tumor purity because of a complex tumor microenvironment, such as mesenchymal IDH wild-type glioblastoma (GBM)^29^, it might introduce a class-specific bias that remains to be investigated systematically. Second, despite a large validation cohort in this study, rare brain tumor types were under-represented or omitted. Thus, ongoing validation in very large multicenter cohorts covering the full spectrum of brain tumors using different techniques is warranted to fully characterize class-specific model performance and identity potential bias.

In conclusion, our study offers a machine learning framework for cross-platform DNA-methylation-based classification of cancer, enabling the development of rapid, resilient, interpretable and accurate diagnostic tests. These methods hold promise to become valuable diagnostic tools for all types of cancer well beyond neuro-oncology.

## Methods

### Patients and materials

This research was carried out in accordance with the Declaration of Helsinki (2013) and approved by the institutional review boards of the Masaryk University Ethical Committee (approval no. 15/2018) and Charité–Universitätsmedizin Berlin (approval no. EA2/041/18). Written informed consent to participate in the study and to the publication of pseudonymized personal data was obtained from all participants before sample processing. Tumor specimens from patients undergoing brain biopsy or surgery for suspected brain tumor underwent (targeted) methylome profiling. Patient age and self-reported sex was recorded where available and is provided in Supplementary Tables 1 and 3. Patient sex was not considered in the study design and any sex bias mainly reflects the epidemiology of specific tumor types. Further information on research design is available in the Reporting Summary linked to this article.

### Methylation microarrays

DNA methylation and copy number analyses were performed using the Infinium Methylation 450k, EPIC and EPICv2 Bead-Chip array platforms (Illumina). All analyses were performed according to the manufacturer’s instructions. Briefly, DNA was extracted from formalin-fixed paraffin-embedded (FFPE) tumor samples using the Maxwell RSC FFPE Plus DNA Purification Kit (Promega Corporation). After bisulfite conversion using the Zymo EZ Methylation Kit (Zymo Research), the Infinium HD FFPE DNA Restore Kit was used for DNA restoration. The beadchips were scanned on the iScan system (Illumina). The unprocessed output data (.idat files) from the iScan reader were checked for general quality measures as indicated by the manufacturer. The .idat files were processed using R/Bioconductor and the minfi package (v.1.36.0) using the preprocessIllumina method^30^.

### WGBS sequencing and processing

Libraries were prepared using the NEBNext Methyl-seq Kit (New England Biolabs) and were then sequenced on an Illumina NovaSeq 6000 platform (cat. no. A01077) at the Berlin Institute of Health Core Unit Genomics over two S4 flow cells in a paired-end setting of 2× 150 bp. Processing of WGBS data from 22 human diffuse GLM samples was performed using the One Touch Pipeline^31^, which uses the Burrows–Wheeler Aligner v.0.6.1 (ref. ^32^) for alignment and methylCtools v1.0.0 (ref. ^33^) for methylation calling. Plus-strand and minus-strand methylated allele frequencies at CpG sites were merged using custom scripts. The mean mapping rate was 99.96% (range: 99.93–99.99%) with 95.7% properly paired (range: 91.2–98.1%) and a 10.2% duplication rate (7.6–13.8%). Alignment resulted in a mean coverage of 70.5 × per sample (range 57–89×).

### Targeted methylation sequencing and processing

Frozen tumor tissues collected during surgery aimed at partial or total tumor resection were used as source material for DNA extraction, which was performed using mechanic homogenization with ceramic beads and subsequent column-based extraction with the DNeasy Blood & Tissue Kit (QIAGEN). Before library preparation, DNA was quantified using the Qubit dsDNA BR Assay Kit (Invitrogen). Sequencing libraries were prepared either with the TruSeq-Methyl Capture EPIC Library Prep Kit (Illumina) or a combination of the SureSelectXT Methyl-Seq Library Preparation Kit with SureSelectXT Human Methyl-Seq target enrichment panel (Agilent Technologies). Sequencing libraries prepared with the TruSeq-Methyl Capture EPIC Library Prep Kit were sequenced on the NextSeq 500 instrument using the NextSeq 500/550 Mid Output Kit v2.5 (150 cycles) (Illumina) in a paired-end setting of 2 × 80 bp. Libraries prepared with the SureSelectXT Methyl-Seq panel were also sequenced on the NextSeq 500 instrument using either the NextSeq 500/550 Mid Output Kit v2.5 (300 cycles) or the NextSeq 500/550 Mid Output Kit v2.5 (150 cycles) in a paired-end setting of 2 × 151 bp and 2 × 80 bp, respectively. Sequencing reads were quality-checked with FastQC v.0.11.9. Adapters and low-quality 3′-end trimming was done with TrimGalore.The alignment to the human reference hg19 genome and methylation calling were carried out completely with Bismark v.0.23.1 (ref. ^34^).

### Nanopore low-pass WGS

A total of 100-400 ng genomic DNA underwent transposase-based library preparation using the Rapid Barcoding Kit (Oxford Nanopore Technologies) according to the manufacturer’s instructions. Libraries were sequenced on R9.4.1 or R10.4.1 flow cells (Oxford Nanopore Technologies) for 6–24 h on a MinION, GridION or PromethION 2 Solo device (Oxford Nanopore Technologies). POD5 or FAST5 raw data were preprocessed using the in-house nanoDx pipeline: after 5mC modified base calling using Dorado (Oxford Nanopore Technologies), reads were aligned to the hg19 reference genome using minimap2 (ref. ^35^) v.2.26; CpG methylation calls were aggregated using modkit (v.0.2.3).

### Feature selection

First, probes that were always methylated or unmethylated across all samples were considered as uninformative and were removed from the dataset.

In the feature processing step, to fill the gap of different sequencing depths, all methylated probes were encoded as 1; correspondingly, unmethylated probes were encoded as −1. To fit the framework to different platforms that may not cover all the 450K CpG sites, masked or undetected features were encoded as 0:

$${\mathrm{CpG}}\; {\rm{site}}=\left\{\begin{array}{l}1,{\mathrm{methylated}}:{\mathrm{beta}}\; {\rm{value}} > 0.6\\ -1,{\mathrm{unmethylated}}:{\mathrm{beta}}\; {\rm{value}}\le 0.6\\\qquad\qquad\quad 0,{\mathrm{missing}}\end{array}\right.$$

### Model training

The brain tumor NN model was trained using 2,801 reference methylomes^8^ generated using Infinium 450K microarrays (Illumina). After binarization of the beta values with a threshold of 0.6 (ref. ^23^) and filtering features with zero variance, 366,263 CpG sites were retained.

To enable the model to make full use of all the information in the features, we sampled features with a fixed sample rate. During model training, in every iteration, samples in the training dataset were randomly masked with the masking rate p, where masked features were encoded as 0. To discover the optimal sample rate, we searched and compared different sample rates via fivefold CV. Finally, the masking rate p = 99.75% was selected.

A normalization function and a softmax layer were used to transform the outputs of the NN into the probabilities of the subtypes of brain tumors. The Adam optimization algorithm was used for training. The model was developed and implemented using PyTorch v.1.13.0 (ref. ^36^).

The pan-cancer model was trained using an in-house assembly of the aforementioned brain tumor reference dataset, the Heidelberg sarcoma reference cohort, nonoverlapping entities from the TCGA and additional single-entity studies (Supplementary Table 2). Methylation data from the TCGA were randomly split into training and validation cohorts at a 2:1 ratio stratified according to tumor type. The TCGA cohort was manually curated in a data-driven approach removing outliers from the *t*-distributed stochastic neighbor embedding projection of the training dataset. After binarization and variance-based filtering of features, 281,232 informative CpG islands were kept for training. The pan-cancer model was then trained for 3,000 epochs using a masking rate p = 99.5%.

### Statistics and reproducibility

The study follows the Data Optimization Model Evaluation principles for the validation of supervised machine learning validation in biology^37^. Training datasets were assembled from public sources and were independent from and nonoverlapping with the validation cohorts. To assemble the pan-cancer training dataset, TCGA data were randomly split into a training and validation dataset at a 2:1 ratio. The training dataset was manually curated in a data-driven approach as follows: *t*-distributed stochastic neighbor embedding mapping of the beta value matrix, colored using the TCGA class labels, was visually inspected and outlier cases were removed manually. All other data sources were integrated without outlier removal and class labels were harmonized across data sources. No statistical method was used to predetermine sample size. To assemble the validation datasets, cases with unclear or unavailable reference diagnosis were excluded from the public and in-house cohorts. Randomization was used to split training and validation cohorts during fivefold CV. When downsampling the training datasets, the process was repeated ten times with different random seeds. Data collection and analysis were not performed blind to the conditions of the experiments. Data distribution was assumed to be normal but this was not formally tested.

### Other analysis

Visualization of genomic information was generated using the R package Gviz^38^. The Python packages seaborn and PyComplexHeatmap were used to plot the heatmaps^39^. CpG sites and genes were annotated using the Python package CpGtools^40^. If not indicated otherwise, the box plots indicate the median, upper and lower quartiles (box limits) and 1.5× the interquartile range (whiskers). The error bars indicate the s.e.m. if not indicated otherwise.

### Reporting summary

Further information on research design is available in the Nature Portfolio Reporting Summary linked to this article.

## Supplementary information

Supplementary Information Code and software checklist.

Reporting Summary

Supplementary Table 1 Supplementary Tables 1–3.

## Source data

Source Data for Figs. 1, 3 and 4 and Extended Data Figs. 1–4. Confusion matrix Fig. 1a. Numerical source data for genomic tracks (Fig. 3e,f). Feature weights (Fig. 3a–d) can be extracted from the model deposited at Zenodo. Confusion matrix Fig. 4c. Source data for Fig. 4d–x are included in Supplementary Table 3. Raw data for the box plots (Extended Data Fig. 1a). Raw data for accuracy over pseudo-time (Extended Data Fig. 1b). Raw data for the circular plot (Extended Data Fig. 2a). Raw data for the box plots (Extended Data Fig. 2b). ROC metrics per fold from 5 × CV. ROC metrics per fold from 5 × CV.

## Data Availability

The targeted methyl-seq raw data have been deposited at the European Genome-phenome Archive (EGA) under accession no. EGAS50000000051. The microarray raw data (accession no. GSE289137) and processed nanopore and WGBS sequencing data (accession no. GSE289246) have been deposited at the Gene Expression Omnibus (GEO). For some sequencing data, no explicit patient consent for the use of genetic data under EU law has been given. In these cases, processed methylation calls (bedMethyl format), sufficient to reproduce all classifications in this work, have been deposited in the GEO. The reference dataset of the Heidelberg brain tumor classifier v11b4 (accession no. GSE90496), containing 2,801 samples, 82 types of brain tumors and nine control classes, was used to train the brain tumor model^8^. The pan-cancer training dataset was assembled from the TCGA, the Heidelberg brain and sarcoma^7^ reference datasets and single-entity studies^41–44^, as detailed in Supplementary Table 2. The beta value matrices of the training datasets and pretrained crossNN models have been deposited at Zenodo (10.5281/zenodo.14006255)^45^. For the validation cohorts, preprocessed public datasets from the following studies were integrated from the sources indicated: MB WGBS^33^ from the International Cancer Genome Project Data Portal release 28 (https://docs.icgc-argo.org/docs/data-access/icgc-25k-data); accession no. GSE142241 for the MB WGBS^46^; accession no. GSE156619 for the ependymoma WGBS^47^; accession no. GSE121721 for the glioblastoma WGBS^48^; accession no. GSE209865 for the nanopore low-pass WGS^23^; and accession no. GSE109379 for the 450K microarray^8^. Methylation data from the TCGA were retrieved via the Genomic Data Commons Data Portal (https://portal.gdc.cancer.gov). Additional nanopore R10.4.1 sequencing data of the primary brain tumors generated from the FFPE specimens were provided by the authors of ref. ^49^. Source data are provided with this paper.

## Code availability

The source code describing the architecture of crossNN, the training of the models and inference is available at https://gitlab.com/euskirchen-lab/crossnn. The nanoDx analysis pipeline implementing the crossNN model for end-to-end analysis of the nanopore sequencing data is available at https://gitlab.com/pesk/nanoDx. A user-friendly graphical user interface (https://crossnn.charite.de) can be used to make predictions from methylomes uploaded as bedMethyl files from several platforms and process methylation microarray IDAT files in real time.
