# Supplementary material for: crossNN is an explainable framework for cross-platform DNA methylation-based classification of tumors
Source: Nat Cancer. 2025 Jun 6;6(7):1283–94. doi: 10.1038/s43018-025-00976-5 (PMC12296554; doi:10.1038/s43018-025-00976-5)
Supplement: Supplementary file 2 — Reporting Summary [file 43018_2025_976_MOESM2_ESM.pdf]

nature portfolio

Corresponding author(s): Philipp Euskirchen

Last updated by author(s): 02/10/2025

Reporting Summary

Nature Portfolio wishes to improve the reproducibility of the work that we publish. This form provides structure for consistency and transparency in reporting. For further information on Nature Portfolio policies, see our Editorial Policies and the Editorial Policy Checklist.

Statistics

For all statistical analyses, confirm that the following items are present in the figure legend, table legend, main text, or Methods section.

n/a	Confirmed	
☐	☒	The exact sample size (*n*) for each experimental group/condition, given as a discrete number and unit of measurement
☐	☒	A statement on whether measurements were taken from distinct samples or whether the same sample was measured repeatedly
☒	☐	The statistical test(s) used AND whether they are one- or two-sided<br>*Only common tests should be described solely by name; describe more complex techniques in the Methods section.*
☒	☐	A description of all covariates tested
☒	☐	A description of any assumptions or corrections, such as tests of normality and adjustment for multiple comparisons
☐	☒	A full description of the statistical parameters including central tendency (e.g. means) or other basic estimates (e.g. regression coefficient) AND variation (e.g. standard deviation) or associated estimates of uncertainty (e.g. confidence intervals)
☒	☐	For null hypothesis testing, the test statistic (e.g. *F*, *t*, *r*) with confidence intervals, effect sizes, degrees of freedom and *P* value noted<br>*Give P values as exact values whenever suitable.*
☒	☐	For Bayesian analysis, information on the choice of priors and Markov chain Monte Carlo settings
☒	☐	For hierarchical and complex designs, identification of the appropriate level for tests and full reporting of outcomes
☒	☐	Estimates of effect sizes (e.g. Cohen's *d*, Pearson's *r*), indicating how they were calculated

Our web collection on statistics for biologists contains articles on many of the points above.

Software and code

Policy information about availability of computer code

Data collection

Raw targeted methylation sequencing data were processed as follows: Sequencing reads were quality checked with FastQC v0.11.9. Adapters and low-quality 3' ends trimming was done with TrimGalore v0.6.10. The alignment to human reference hg19 and methylation calling were carried out completely with Bismark v0.23.1. Processing of WGBS data from 22 human diffuse glioma samples was performed using the One Touch Pipeline (OTP) which uses bwa v0.6.1 for alignment and methylCtools v1.0.0 for methylation calling. Methylation microarrays were preprocessed using R/Bioconductor and the minfi package (version 1.36.0). Nanopore POD5 or FAST5 raw data were preprocessed using the in-house nanoDx pipeline: after 5mC modified basecalling using dorado (Oxford Nanopore Technologies, UK), reads were aligned to the hg19 reference genome using minimap2, version 2.26, and CpG methylation calls were aggregated using modkit (version 0.2.3).

Data analysis

Source code describing the architecture of crossNN, training of models and inference is available at https://gitlab.com/euskirchen-lab/crossnn. The nanoDx analysis pipeline implementing the crossNN model for end-to-end analysis of nanopore sequencing data is available at https://gitlab.com/pesk/nanoDx. A user-friendly graphical user interface (https://crossnn.charite.de) allows to make predictions from methylomes uploaded as bedMethyl files from various platforms and process methylation microarray IDAT files in realtime.
The model was developed and implemented using PyTorch 1.13.036. Visualization of genomic information was generated by R package Gviz. Python package seaborn and PyComplexHeatmap were used for plotting heatmaps. CpG sites and genes were annotated using Python package CpGtools.

For manuscripts utilizing custom algorithms or software that are central to the research but not yet described in published literature, software must be made available to editors and reviewers. We strongly encourage code deposition in a community repository (e.g. GitHub). See the Nature Portfolio guidelines for submitting code & software for further information.

Data

Policy information about availability of data

All manuscripts must include a data availability statement. This statement should provide the following information, where applicable:

- Accession codes, unique identifiers, or web links for publicly available datasets
- A description of any restrictions on data availability
- For clinical datasets or third party data, please ensure that the statement adheres to our policy

Targeted methyl-seq raw data have been deposited at the European Genome-phenome archive (EGA) under accession no. EGAS50000000051. Microarray raw data (GSE289137) and processed nanopore and WGBS sequencing data (GSE289246) have been deposited at Gene Expression Omnibus (GEO). For some sequencing data, no explicit patient consent for deposition of genetic data under EU law has been given. For these cases, processed methylation calls (bedMethyl format), sufficient to reproduce all classifications in this work, have been deposited in GEO.
The reference set of the Heidelberg brain tumour classifier v11b4 (GSE90496) containing 2,801 samples and 82 types of brain tumors and 9 control classes was used for brain tumor model training. The pan-cancer training set was assembled from The pan-cancer training set was assembled from The Cancer Genome Atlas, the Heidelberg brain and sarcoma reference sets and single entity studies as detailed in Supplementary Table 2. Beta values matrices of the training sets and pre-trained crossNN models have been deposited at Zenodo (https://doi.org/10.5281/zenodo.14006255).
For validation cohorts, preprocessed public datasets from the following studies were integrated from the sources indicated: medulloblastoma WGBS from the International Cancer Genome Project Data Portal release 28 (https://docs.icgc-argo.org/docs/data-access/icgc-25k-data), GSE142241 for medulloblastoma WGBS, GSE156619 for ependymoma WGBS48, GSE121721 for glioblastoma WGBS, GSE209865 for nanopore low-pass WGS, GSE109379 for 450K microarray. Methylation data from The Cancer Genome Atlas were retrieved via the GDC Data Portal (https://portal.gdc.cancer.gov). Additional nanopore R10.4.1 sequencing data of primary brain tumors generated from FFPE specimens were kindly provided by the authors of Afflerbach et al..

Research involving human participants, their data, or biological material

Policy information about studies with human participants or human data. See also policy information about sex, gender (identity/presentation), and sexual orientation and race, ethnicity and racism.

Reporting on sex and gender	Patient sex was recorded where available. Sex or gender aspects do not apply within the scope of this study.
Reporting on race, ethnicity, or other socially relevant groupings	We did not take into account race, ethnicity, or other socially relevant groupings in this study.
Population characteristics	The validation cohort comprises adult and pediatric patients (N=5379, brain tumor subset only N=2090) diagnosed with neoplasms of different tumor types and organ sites. Mean patient age is 52.7 years (range 0 - 90 years). 44.5% of patients were female. Sex information was based on annotation of clinical records (self-reported).
Recruitment	For samples sequenced prospectively, patients undergoing neurosurgery for suspected brain tumor were recruited to the study. Patient registration was performed preoperatively in order to minimize selection bias. Yet, requirement for informed consent of the patient or legal guardian possibly introduced selection bias. For public and retrospective data, data was selected on availability. Bias with respect to the epidemiology of brain tumors includes enrichment for pediatric and rare tumors while the cohort is still not representative for very rare entities.
Ethics oversight	The research was carried out in accordance with the Declaration of Helsinki and approved by the institutional review boards at Masaryk University Ethical Committee (approval no. 15/2018) and Charité – Universitätsmedizin Berlin (approval no. EA2/041/18).Informed consent to participate in the study and to publication of pseudonymized personal data was obtained from all subjects prior to sample processing.

Note that full information on the approval of the study protocol must also be provided in the manuscript.

Field-specific reporting

Please select the one below that is the best fit for your research. If you are not sure, read the appropriate sections before making your selection.

☒ Life sciences ☐ Behavioural & social sciences ☐ Ecological, evolutionary & environmental sciences

For a reference copy of the document with all sections, see nature.com/documents/nr-reporting-summary-flat.pdf

Life sciences study design

All studies must disclose on these points even when the disclosure is negative.

Sample size	Sample size of the training sets and validation cohort was dictated by the availability of in-house and public datasets. They are identical or superior to current landmark studies (Capper et al., Nature 2018; Koelsche et al., Nat Comm 2021 and others) as we integrated their publicly available data.
Data exclusions	Data without available reference diagnosis ("ground truth"), cases with tumor entities not in the reference set and data sets with less than

Data exclusions	1000 informative CpG sites were excluded from analysis. The minimum CpG limit has been pre-established in Kuschel et al., Neuropathol Appl Neurobiol 2023.
Replication	Random splitting of TCGA data for building the pan-cancer training set was performed once. All downsampling experiments were repeated 10 times with different random seeds. The brain and pan-cancer crossNN model were each validated in an independent validation cohort once to avoid overfitting.
Randomization	TCGA methylation data were randomly split into training (2/3) and validation (1/3) cohort. 5fold cross validation was performed for internal validation of classifiers.
Blinding	Assembly of the validation set was performed before and without knowledge about crossNN classification results. Model validation in independent cohorts was performed after final training of the model.

Reporting for specific materials, systems and methods

We require information from authors about some types of materials, experimental systems and methods used in many studies. Here, indicate whether each material, system or method listed is relevant to your study. If you are not sure if a list item applies to your research, read the appropriate section before selecting a response.

Materials & experimental systems

n/a	Involved in the study
☒	☐ Antibodies
☒	☐ Eukaryotic cell lines
☒	☐ Palaeontology and archaeology
☒	☐ Animals and other organisms
☒	☐ Clinical data
☒	☐ Dual use research of concern
☒	☐ Plants

Methods

n/a	Involved in the study
☒	☐ ChIP-seq
☒	☐ Flow cytometry
☒	☐ MRI-based neuroimaging

Plants

Seed stocks	*Report on the source of all seed stocks or other plant material used. If applicable, state the seed stock centre and catalogue number. If plant specimens were collected from the field, describe the collection location, date and sampling procedures.*
Novel plant genotypes	*Describe the methods by which all novel plant genotypes were produced. This includes those generated by transgenic approaches, gene editing, chemical/radiation-based mutagenesis and hybridization. For transgenic lines, describe the transformation method, the number of independent lines analyzed and the generation upon which experiments were performed. For gene-edited lines, describe the editor used, the endogenous sequence targeted for editing, the targeting guide RNA sequence (if applicable) and how the editor was applied.*
Authentication	*Describe any authentication procedures for each seed stock used or novel genotype generated. Describe any experiments used to assess the effect of a mutation and, where applicable, how potential secondary effects (e.g. second site T-DNA insertions, mosiacism, off-target gene editing) were examined.*
